# Health professionals’ sentiments towards implemented information technologies in psychiatric hospitals: a text-mining analysis

**DOI:** 10.1186/s12913-022-08823-4

**Published:** 2022-11-28

**Authors:** C. Golz, S. Aarts, C. Hacking, S. Hahn, S.M.G. Zwakhalen

**Affiliations:** 1 Department of Health Professions, Bern University of Applied Sciences, Murtenstrasse 10, 3011 Bern, Switzerland; 2 Department of Health Services Research, Maastricht University, Maastricht, The Netherlands; 3 Living Lab in Ageing and Long-Term Care, Maastricht University, Maastricht, The Netherlands

**Keywords:** Text-Mining, Information Technology, Sentiments, Psychiatric Hospital, Health Professional

## Abstract

**Background:**

Psychiatric hospitals are increasingly being digitalised. Digitalisation often requires changes at work for health professionals. A positive attitude from health professionals towards technology is crucial for a successful and sustainable digital transformation at work. Nevertheless, insufficient attention is being paid to the health professionals’ sentiments towards technology.

**Objective:**

This study aims to identify the implemented technologies in psychiatric hospitals and to describe the health professionals’ sentiments towards these implemented technologies.

**Methods:**

A text-mining analysis of semi-structured interviews with nurses, physicians and psychologists was conducted. The analysis comprised word frequencies and sentiment analyses. For the sentiment analyses, the SentimentWortschatz dataset was used. The sentiments ranged from -1 (strongly negative sentiment) to 1 (strongly positive sentiment).

**Results:**

In total, 20 health professionals (nurses, physicians and psychologists) participated in the study. When asked about the technologies they used, the participating health professionals mainly referred to the computer, email, phone and electronic health record. Overall, 4% of the words in the transcripts were positive or negative sentiments. Of all words that express a sentiment, 73% were positive. The discussed technologies were associated with positive and negative sentiments. However, of all sentences that described technology at the workplace, 69.4% were negative.

**Conclusions:**

The participating health professionals mentioned a limited number of technologies at work. The sentiments towards technologies were mostly negative. The way in which technologies are implemented and the lack of health professionals’ involvement seem to be reasons for the negative sentiments.

**Supplementary Information:**

The online version contains supplementary material available at 10.1186/s12913-022-08823-4.

## Introduction

The increasing possibilities through technological innovations and their expected benefits have accelerated the digital transformation in health care [1]. This increasing use of and reliance on digital transformation in health care is underlined by research [2]. Marques and Ferreira [2] highlighted that there has been an exponential increase in studies over the last decade, with a focus on exploring the potential of technological solutions to improve the quality and safety of health care. However, the majority of studies included were conducted in the acute medical care setting. This indicates an imbalance in the research conducted into digital transformation process across the different health care sectors.

The mental health care setting is just at the beginning in the digitalisation of patient care or of the administrative processes [3]. Developers and researchers often fail to develop, implement, and evaluate Information Technology (IT) in mental health care mainly due to barriers in engagement, effectiveness, equity, access, ethical concerns and concerns of worsening the therapeutic relationship [4–8]. Furthermore, missing infrastructure (e.g. no suitable devices or Wi-Fi) as well as insufficient skills of health professionals hamper successful implementations [5]. IT is defined as the ‘application of information and communication technologies tools including computer network, software and hardware required for internet connection ‘ [9].

The expected advances of technological solutions like artificial intelligence, wearables, e-health or standardised data formats through electronic health records are seen as the promotors of the future of digital psychiatry [3, 8, 10]. Despite the difficulties in the development and implementation of some digital technologies, advantages of already implemented technologies could be identified. For example, the use of electronic health record in the mental health care setting led to a significant increase of timely access and availability of patient information for the health professionals [11]. Furthermore, the implementation of telemental health led to enhanced accessibility of the services – of equivalent therapeutic quality – for immobile patients or patients living in rural regions [12, 13]. However, the use of technology resulted in several adverse effects among the health professionals working in the mental health care setting, such as higher burnout-symptoms, increased intention to leave the organisation or physical stress reactions [14].

One reason for adverse effects of technologies at work on health professionals’ health is the lack of attention to the health professionals’ attitude (e.g. anxiety, uncertainty) towards technologies during the development and introduction of technologies at work [15]. A positive attitude towards technology usage is associated with reduced technology-related stress [16], which in turn is a relevant influencing factor on multiple health-related outcomes among health professionals [14]. Attitudes are based on a feeling about a situation or a way of thinking about something – expressed by individuals verbally, in writing or in gestures – and are the sentiment of this person [17, 18]. Sentiments can be either negative, neutral or positive [19]. In this context, sentiments can describe the feelings towards technology or the way of behaving when interacting with technology at work.

So far, research on health professionals’ sentiments towards technology in psychiatric hospitals is limited [20]. However, a more in-depth understanding of health professionals’ sentiments may give a better insight into their feelings towards technology.

The aim of this study, therefore, was (a) to identify the implemented information technologies in psychiatric hospitals and (b) to describe the health professionals’ sentiments towards technologies.

## Methods

A text mining analysis of semi-structured interviews to describe health professionals’ sentiments about already implemented technologies in psychiatric hospitals was conducted. Text mining is an umbrella term for computational processes to analyse unstructured text data [21]. Within the text mining, the data pre-processing and analysis is automated, which enables the identification of new information and relationships within comprehensive unstructured datasets [21]. The text mining approach can be used to count word frequencies and to identify patterns or sequences of used words, as well as sentiment analysis. Sentiment analysis is a text mining method that quantifies the emotional value in a text [18]. It is an objective and reproducible way of assigning a number about how negative or positive a piece of text is. Text mining has been recognised in health science as a suitable method to extract information from electronic health records [22] or from transcripts of single or focus group interviews [23–25].

### Study sample

A convenience sample of nurses, physicians and psychologists working in psychiatric hospitals was considered. The study was first presented to the management of three psychiatric hospitals in the German-speaking part of Switzerland, two of which thereupon indicated their interest in participating in the study (one private and one public psychiatric hospital each). After managerial decision to take part in the study, the management of each psychiatric hospital provided an internal coordinator to assure adequate information provision. The internal coordinator confirmed that technologies are applied in the workplace. The internal coordinators were either the medical director or the person responsible for the nursing development of the participating psychiatric hospital. They were asked to provide the employed health professionals with an informative letter about the study and to invite them to participate in the interviews. Physicians, nurses and psychologists subsequently contacted the researcher directly if they were interested in participating. To meet the inclusion criterion, participants had to have been employed by the current employer for at least 1 year, in order to ensure that these professionals had had sufficient experience with technology in their work.

### Data collection

Data were obtained in semi-structured individual interviews between June 2020 and March 2021 in person using an interview guide. An interview guide is defined as ‘a list of questions, which directs conversation towards the research topic during the interview’ [26]. Its’ form is considered ‘loose’ and ‘flexible’ with topics, covering the main topics of the research subject [26]. The interview guide (Multimedia Appendix A) was developed based on the technology acceptance model [27]. This model describes the influence of attitude on the behavioural intention to use an IT [27]. The used interview guide covers the determinants of the dimensions from the model, such as ‘perceived usefulness’ (e.g. How does the [technology] influence your performance?), ‘perceived ease of use’ (e.g. How do you assess your competence in dealing with digital technologies in your workplace?) and ‘computer anxiety’ (e.g. How do you experience the overload caused by digital technologies in your work?). It also covers the moderators of the dimensions, as they influence the ‘behavioural intention’ of the user [27]. We included questions for the following moderators in the interview guide:

‘Experience’ (e.g. What digital technology has been implemented recently and how did you experience this implementation?), ‘management / organizational support (e.g. How do you experience the change in your role due to digital technologies?), ‘design characteristics (e.g. How do you feel about the possibility of another person being able to monitor all your performance through a digital technology?) and ‘user participation’ (e.g. What digital technologies would you like to have to better manage your work?). These aspects form the individual’s attitude towards the technology, which leads to the intention to use or non-use of the technology [19]. The interviews were conducted in Swiss German and audio recorded with a recording device after written consent of the participants.

### Data analysis

#### Transcription and translation

The audio files were transcribed verbatim by one researcher, to textualize them as unstructured data in interview transcripts [28]. The Swiss German (a spoken German dialect with no direct written equivalent) was translated into the German language by one research member with German as native language and cross-checked by another research member with Swiss German as native language. In this process, the translations were also checked for correct spelling, to meet the requirements for data pre-processing, which is based on German vocabulary (e.g. ‘*practical’* [gäbig bzw. praktisch]).

#### Data pre-processing

The interview transcripts were pre-processed and analysed by using the statistical software R version 4.0.4 with Studio 1.4.1106 [29] with the following packages: spacyr [30], tm [31] and tidytext [32]. The data pre-processing comprised several steps: (a) deletion of the interviewers’ text from the transcripts – that is, transcribed questions and statements of the interviewer. (b) The unstructured text data were transformed into a list, where each word was placed in one row. This process is referred to as tokenisation [32]. (c) The words were reduced to their dictionary root (base form) by using the spacyr package [30] with the German-language-specific package ‘de_core_new_lg’. Word forms with the same root, such as ‘*makes*’ [macht], ‘*made*’ [gemacht] and ‘*make*’ [mache] are aggregated in the basic form ‘*to make*’ [machen]. This process is known as lemmatisation [21]. (d) Umlauts (ä, ö, ü) were transformed to (ae, ou, ue). Stopwords (e.g. I, and, it) predefined in the package tm [31], numbers, punctuation marks and other words not relevant for the analysis (e.g. names, greetings) were deleted.

#### Frequency and sentiment analysis

After data pre-processing, frequencies of the mentioned technologies and the sentiments using the ‘*SentimentWorschatz*’ (SentiWS) [33] were calculated. The sentiment analysis quantified the attitudes, opinions and emotions of the participants towards the technologies [18]. The current version of SentiWS consists of 1650 positive sentiments in their basic word form and 1800 negative sentiments in their basic word form. The sentiments’ values are interval-scaled and range between -1 (strongly negative) and 1 (strongly positive) [33]. For example, the word ‘*great*’ [super] has a positive polarity with a value of 0.5012 and the word ‘*bad*’ [schlecht] has a negative polarity with a value of -0.7706. To avoid misclassification of sentences with negation, the identified sentiments were screened for their potential relation with the words ‘not’ [nicht] and ‘no/none’ [kein]. Sentiments with a negation in the sentence were recoded accordingly and added to the SentiWS with reversed polarity [34], for example ‘not bad’ [nicht schlecht] with a value of 0.7706. The identified sentiments were used for three different analyses. (a) For the first analysis, the means of words that describe negative $({\mu }_{neg})$ or positive $({\mu }_{pos})$ sentiments were calculated to compute the average proportion of negative sentences about technologies at work per interview and across all interviews $({\mu }_{neg} /$ ${\mu }_{pos}- {\mu }_{neg})$. (b) For the second analysis, the frequency of the sentiments per technology was calculated and multiplied with the sentiments’ value from the SentiWS. The relation between sentiment value and frequency emphasises that a few sentiments with a higher value have a stronger impact on the quantified attitude towards a specific technology than many sentiments with a low value. (c) As a third analysis, n-gram (*n* = 5) analysis of sentences describing sentiments per technology was conducted for a better understanding of the context in which a sentiment has been mentioned. The n-gram analysis is a sequence of n elements from a given text. The n elements are in the word order close to a defined keyword in the text, where the keyword is also one word of the n elements. Analysis was conducted in an iterative process in which new interview transcripts were added sequentially to evaluate when data saturation was achieved [35].

#### Reporting and visualisation

For credibility, preliminary findings and interpretations were checked and discussed within the research team. For dependability, the data analysis was audited by two co-researchers. Furthermore, replicability was enabled through the provision of the statistical software script file [36]. The script file is available as Multimedia Appendix B. The visualisation was conducted by using the package ggplot2 [37]. The frequencies of the mentioned technologies have been displayed in table form and for the sentiments in a bar chart. The average proportions of negative sentences were visualised in a scatterplot with one point per interview transcript and the average across all interview transcripts. The results of the sentiment analyses for each identified technology were visualised in a bar chart, displaying how often a sentiment related to a technology for all interviews.

## Results

In total, 20 health professionals participated in the study: 11 nurses (55%), 5 physicians (25%) and 4 psychologists (20%). Most of the participants were female (*n* = 16; 80%) and the mean age was 39 years (SD = 13.05 years). The mean duration of a single interview was 42 min (SD = 7.89 min).

The keyword density per technology ranged between 0.32% and 0.01% in the interview transcripts. The overall density of mentioned technologies in the interview transcripts was 1%. The health professionals mentioned hardware and software when asked about technologies they used. In the interviews, the participants mainly talked about the computer (28%), followed by the phone (18%) as the hardware. Regarding software, the majority of the participants talked about email using Microsoft Outlook (22%), followed by the electronic health record (18%; Table 1).

**Table 1:** Hardware and software that was mentioned in the interviews ordered by frequency

	**Information Technology**	**Frequency of mentioning, n (%)**
**Hardware**	Computer	203 (28)
	Phone	130 (18)
	Laptop	52 (7)
	Electrocardiogram	12 (1.7)
	Voice recorder	2 (0.3)
**Software**	Email	161 (22)
	Electronic health record	129 (18)
	Shift planning tool	14 (2)
	WhatsApp	11 (2)
	Wi-Fi	6 (1)

### Sentiment analysis

**Fig. 1:**
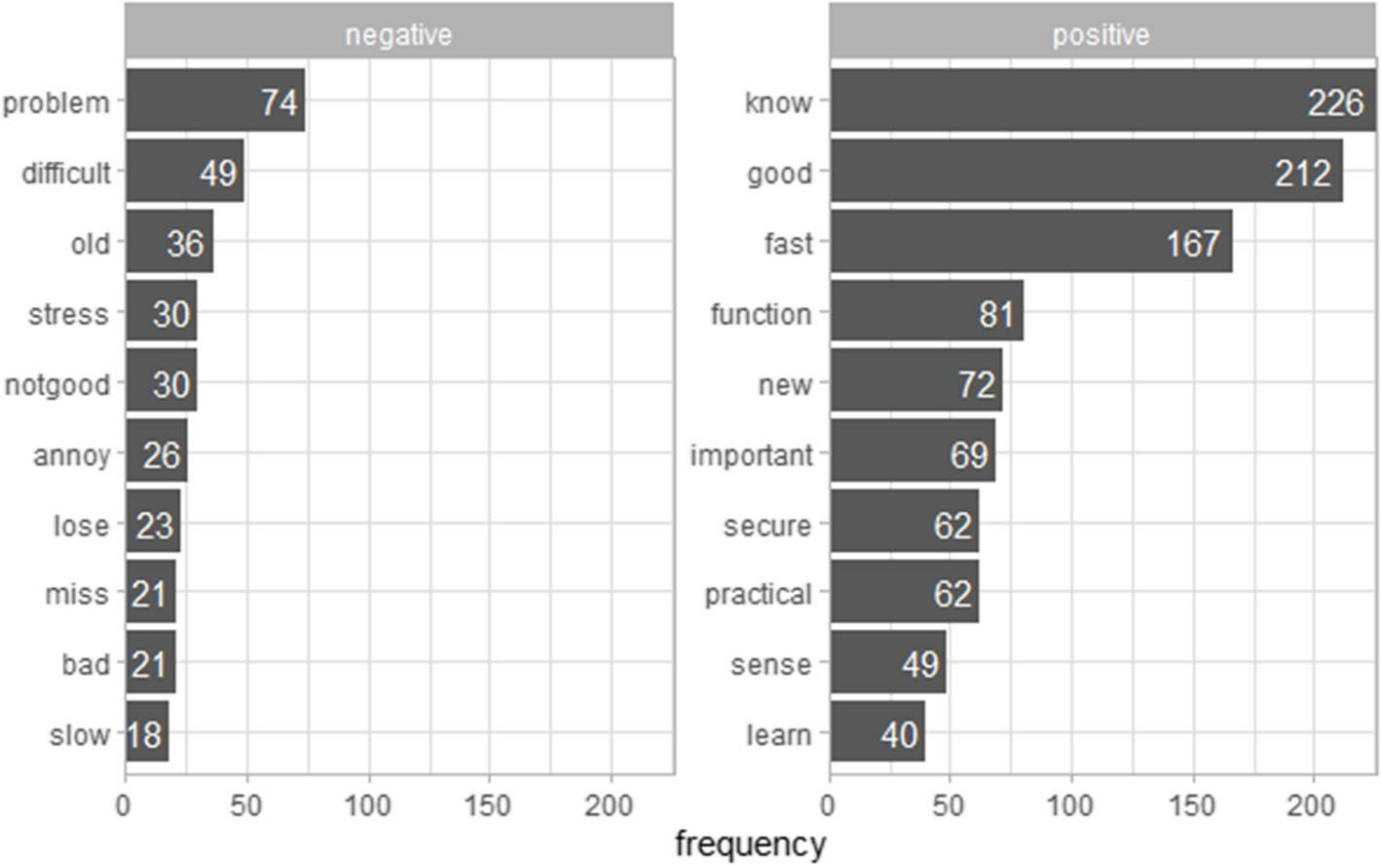
SentiWS sentiments from the interviews ordered by their frequency. The left bar chart displays the sentiments with a negative polarity. The right bar chart displays the sentiments with a positive polarity

Overall, 4% of the words in the transcripts had a non-zero positive or negative connotation. The remaining words were identified as neutral. The majority of words with a non-zero sentiment were identified as positive (73%). The most frequently used word with a positive polarity was ‘*know*’ [wissen] (11%), followed by ‘*good*’ [gut] (10%) and ‘*fast*’ [schnell] (8%). The most frequently used word with a negative polarity was ‘*problem*’ [Problem] (8%), followed by ‘*difficult*’ [schwierig] (5%) and ‘*old*’ [alt] (4%) (Fig. 1).

**Fig. 2:**
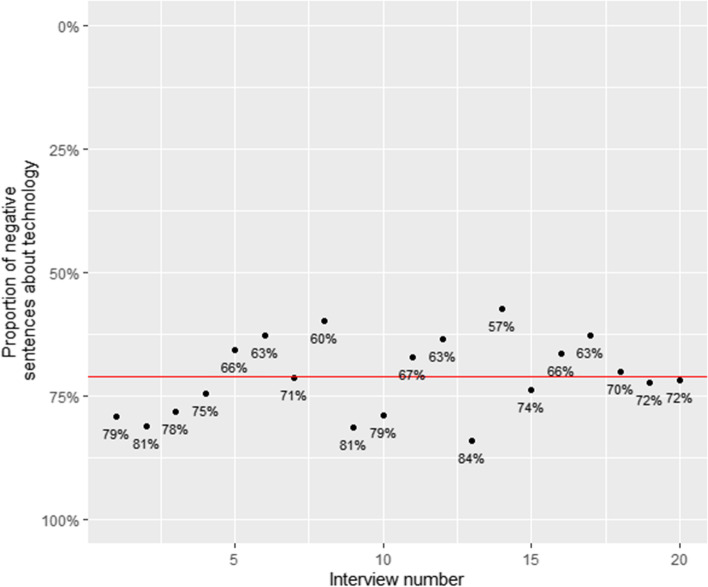
Average proportion of negative sentences about technology at work. The horizontal line indicates the average proportion of all interviews. The points indicate the average proportion per interview

The majority of the identified words with a non-zero sentiment indicated small values on the polarity from -1 (negative) to 1 (positive). The overall mean value for the positive sentiments was 0.11 and the mean value of the negative sentiments was -0.26. There was a negative sentiment towards technologies among the participants. The average proportion of negative sentences about technology at work in the transcripts was 69.4% (SD = 7.73%) (see Fig. 2).

For several technologies (i.e. shift planning tool, WhatsApp, voice recorder and electrocardiogram), the sentiment analysis did not yield statistically significant results because these were hardly mentioned by the participants and therefore not related with any sentiment (see Table 1). The participants mentioned positive and negative properties for the electronic health record, computer, phone, email and laptop. The participants perceived computer work mostly negatively. This can be seen from the fact that although more positive sentiments were used in the context of the word ‘*computer*’, negative sentiments clearly outweighed positive sentiments in terms of polarity. The participants used the word ‘*computer*’ in the context of the positive sentiments ‘*fast*’, ‘*practical*’ and ‘*integrative*’ [schnell, praktisch and integrieren] but also in the context of the negative sentiments ‘*old*’, ‘*problematic*’, ‘*not good*’, ‘*unfortunately*’, ‘*destroy*’ and ‘*burden*’ [alt, problematisch, nicht gut, leider, vernichten and belasten, respectively].

**Fig. 3:**
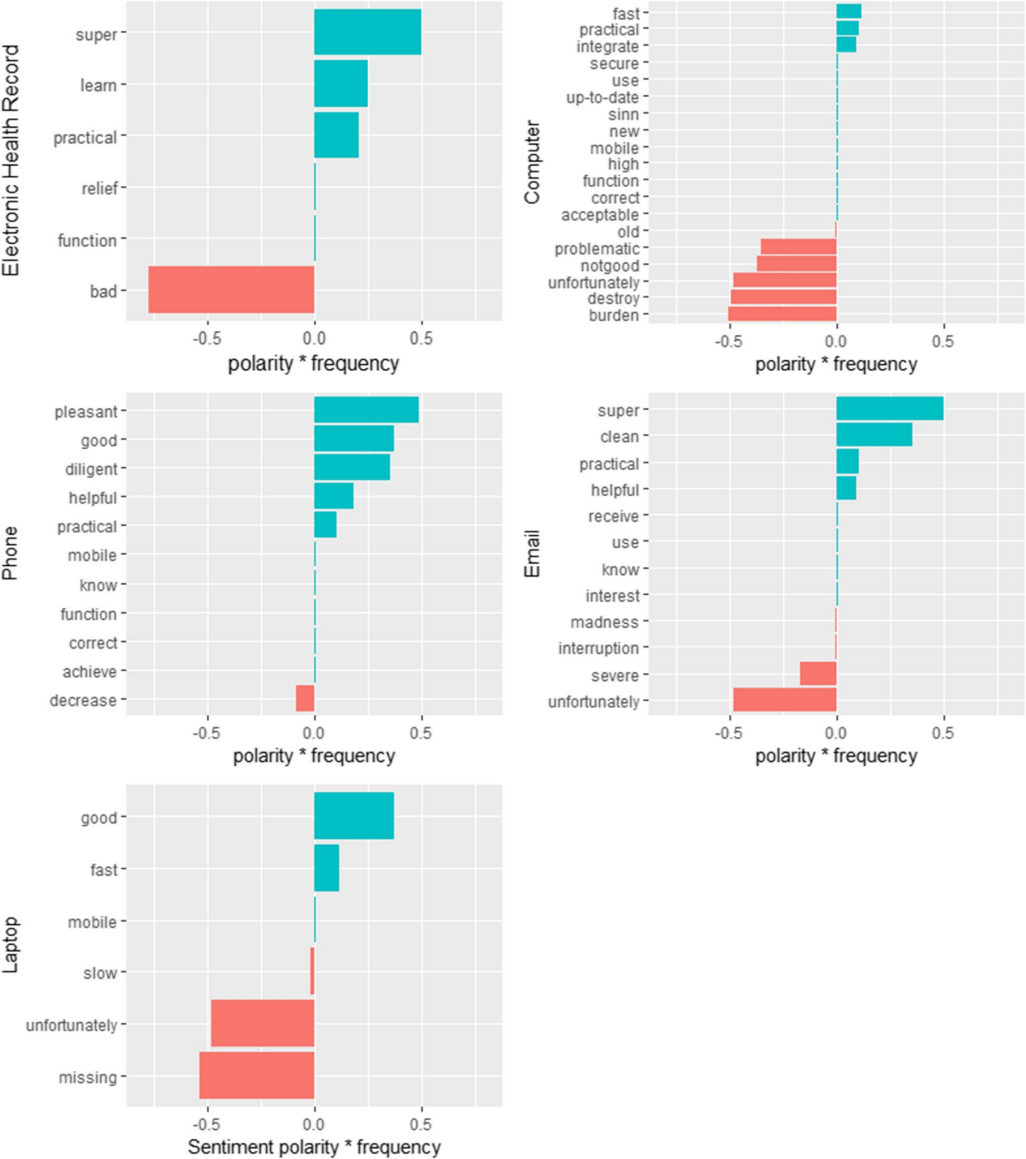
Results of the sentiment analyses for each technology with identified sentiments. The X-axis displays the frequency of the sentiments multiplied by the sentiments’ polarity from the SentiWS. The Y-axis displays the sentiments

Figure 3 summarises the sentiment analyses for the above-described technologies with their related sentiments. The polarity multiplied by the frequency of the sentiments highlighted that a few sentiments with a higher value have a stronger impact on the quantified attitude towards a specific technology than many sentiments with a low value.

The n-gram analysis for words in their consecutive order related to a keyword revealed that although the participants could use ‘*laptops*’, the devices, unfortunately, needed to be ‘*connected to the power*’ or the ‘*internet*’ due to ‘*weak batteries*’ or ‘*missing Wi-Fi*’ options at work. The work with the ‘*phone*’ was experienced mainly as positive. However, answering the phone while being occupied in a conversation with a patient was mentioned as an ‘*avoidable interruption*’. As indicated in Fig. 3, ‘*writing email*’ was a frequent activity among the participants, which predominantly was associated with positive sentiments. On the other hand, the health professionals experienced an ‘*overload*’ of emails and an ‘*interruption*’ of their work.

## Discussion

The current study focused on identifying the implemented technologies in psychiatric hospitals and exploring the health professionals’ sentiments towards technologies. The results showed that computer, phone and email were used at work. Findings showed that the participants at the same time had both positive and negative sentiments towards each discussed technology. The majority of the identified sentiments were rather negative regarding technologies at work. The findings underline the influence of the user’s experience on the attitude towards using technology, as demonstrated in the technology acceptance model [27]. In our study, the five topics ‘Job Relevance’, ‘Output Quality, ‘, ‘User participation’, ‘Management support’ and ‘Organisational support’ from the technology acceptance model derived, acknowledging their statements based on their experience.

### Job relevance & output quality

Despite being aware of the positive attributes of the technologies, health professionals reported being confronted with issues during the interaction with technology at work that led to rather negative sentiments towards them. The duality of the sentiments towards technologies – that one sees the benefits but cannot take full advantage of them due to barriers – is consistent with the existing literature regarding health professionals’ attitudes towards information systems [38, 39]. Job relevance and output quality are known to have an interactive effect on perceived usefulness [27]. In this context, health professionals seem to believe that technology is an added value for their work. However, the inadequate output quality leads to an overall negative attitude toward technology at work.

In our study the negative sentiments regarding, for example, the technology ‘*laptops*’ were mentioned in the context of lack of mobility due to lack of Wi-Fi or because of the fast battery discharge. This shows that the negative sentiments regarding ‘*laptop*’ were related to the quality of the technology or the connectivity and less to the fact that technology is used. Thus, it might be argued that health professionals have positive sentiments towards technologies in general, but this positivity is diminished by a lack of user-friendliness or expediency.

### User participation

In a recent study, nurses perceived electronic health records as supportive during the provision of care, but they also rated the user-friendliness as low [40]. One reason for the low user-friendliness might be the lack of attention to the evaluation of technologies during development and implementation alongside the health professionals [41]. In this study, the negative sentiment ‘*unfortunately*’ [leider] was mentioned in connection with various technologies. ‘*Unfortunately*’ in this context can be interpreted as a regret or a disappointment of the participant that the technology does not meet the expectations. User involvement in the development and evaluation of technologies often starts too late [41], so this discrepancy between expectation and experience cannot be given adequate attention. This might have contributed to the fact that negative sentiments towards technologies outweighed positive ones. The involvement and contribution by health professionals to technology that is useful at work should serve as the basis to reduce the health professionals’ reluctance towards technology, which might be the reason why digitalisation is progressing more slowly in mental health than in other health settings [5–7].

### Organizational support

Poor battery life and weak Wi-Fi could underline the findings that the IT-departments are insufficiently involved in the implementation processes of technological innovations [42]. The IT departments of health organisations have reported several barriers for successful implementation of technology: a lack of resources, the absence of 24/7 IT services for health professionals and not being involved in technology-related decisions by the management [42]. Our findings suggest that before the digital future of psychiatry can be pondered [43], technical requirements must be met. For example, if wearables should be implemented to measure patient data [3], a reliable Wi-Fi for data transmission is crucial.

Psychiatric hospitals are acknowledged to be just at the beginning of the most innovative and potentially disruptive changes through digital transformation [3]. To master this expected change in the long term, the mostly negative sentiments towards technology among health professionals must be converted to positive sentiments [7].

### Management support

To achieve this change, decision-makers in psychiatric hospitals need to be committed and assess the health professionals’ needs of technologies, in particular the functionality and suitability for everyday use. For this endeavour, they should involve health professionals early in the development and implementation process [41] and learn from their point of view towards the technologies at hand. Theoretical models, such as the technology acceptance model 3 [27], should be used as a foundation in order to understand the systemic connectedness of factors, which influence the sustainable use of technology at work.

### Implications for practice

Not all aspects from the technology acceptance model emerged from our findings. One reason could be that statements are made in interviews that affect several aspects of the theoretical construct equally and overlap. For an overall understanding of the attitudes towards technology, a complementary quantitative approach based on the TAM3 would be suitable. However, we found that, in particular, the ‘user participation’, ‘management support’ and ‘organizational support’ are seen as relevant by the health professionals.

The model highlights that user experience highly influences all aspects of intention to use a technology. Bourla, Ferreri [20], for example, indicated that psychiatrists’ resistance to technology is due to fear of loss of control because of missing involvement and knowledge. To achieve the supportive effect of digitalisation for health professionals, the technologies must function according to the health professionals’ expectations. In addition, health professionals must be trained in the usage of these technologies. Also, guidelines for using technologies at work must be made available to the health professionals [44]. For example, the guideline for work-specific emails within the organization should define, which information should be sent to who, during which time slot and who should be in carbon copy (cc). With regard to the phone, the guideline should define, during which tasks a forwarding of the phone is allowed and for which questions one reports to the responsible person by phone. Such clarification should lead to a reduction of interruption at work [44].

### Strengths and limitations

One strength of this study is that it has given the health professionals’ a voice regarding their experiences with technology at work. The results highlight that the health professionals have a clear attitude towards technologies but that those attitudes are not being met accordingly. Moreover, trustworthiness has been established by aiming for credibility, dependability and confirmability [45]. Researchers and data managers of health organisations can use the script file to conduct projects with comparable aims without the need for major adjustments of the data pre-processing and analysis. The data set can be extended by additional transcripts without additional effort, or the analysis can be re-evaluated with new transcripts on a recurring basis. Confirmability was extended by reducing the researcher’s influence on the result by replacing part of the manual work by systematic computational processes.

The current results should also be viewed in terms of some limitations. One limitation of the study is the number of transcripts included. No generalizability is possible due to small sample size. However, data saturation was reached, since no new topics regarding implemented technologies at work emerged by increasing in the number of interview transcripts in the analysis [35]. Furthermore, a recent systematic review on minimum sample size for data saturation in qualitative research concluded that 9 – 17 interviews were found to be sufficient to reach data saturation, which was met in this study [46]. Nevertheless, text mining is known for the analysis of comprehensive data sets that are too large to be analysed manually [36]. A few technologies could not be sufficiently related to sentiments because they were rarely mentioned in the transcripts used. Increasing the number of interviews could have provided further insights regarding the health professionals’ sentiments towards technologies at work. However, regarding the mentioned data saturation, it is not granted that more interviews would allow other technologies to be linked to sentiments. Another limitation lies in the data pre-processing. Data pre-processing of unstructured German text data is limited to the available software packages. The authors of the spacyr package used for the lemmatisation reported an accuracy of 73% for this process, which led to words that have not been or incorrectly lemmatised [30]. These errors had to be corrected manually and will differ from other data sources. Moreover, the SentiWS does not allow automatic detection of sentences with negation. Although this was considered in our data pre-processing [34], it bears the risk of not having identified all negated statements as such. Also, the SentiWS does not include all sentiments of German language but is being updated continuously [33]. However, with regard to comparable lexicons, the SentiWS showed better performance in terms of identifying sentiments [47]. Some of the questions from the interview guide were negatively phrased, in particular, those focussing on Computer Anxiety. Albeit the determinant elaborates the anxiety towards technology usage, negative formulated questions might have influenced the interviewees’ tendency. Furthermore, it cannot be excluded that a sampling bias is present. The convenience sampling approach could have introduced some bias that people who are already sensitised to the topic and are interested in expressing their views are more likely to participate. The slight tendency to make negative statements about technologies and the identification of positive and negative properties, however, suggests that no extreme opinions were represented in this sample.

## Conclusions

This project has highlighted that behind a positive or negative attitude towards technologies, there can be a tension between desired added value and experienced disadvantages. Nurses, physicians and psychologists in psychiatric hospitals mentioned a limited number of technologies at work, with the computer, documentation in the electronic health record and communication via email being the most discussed technologies. The results indicate that the current technologies do not meet the health professionals’ expectations. Future research should focus on implementation studies including health professionals’ sentiments to identify important factors for a successful implementation. Health professionals should be involved early in the development process, and research should support psychiatric hospitals in this process from development to evaluation of digital solutions at work.

## Supplementary Information

**Additional file 1.**

**Additional file 2.**

## Data Availability

The datasets generated and/or analyzed during the current study are not publicly available due to potentially patient identifiable information as part of data. Anonymized data supporting the findings of this study are available from the corresponding author Christoph Golz, christoph.golz@bfh.ch. The script file and interview guide are available as supplement.
