# Supplementary material for: Health professionals’ sentiments towards implemented information technologies in psychiatric hospitals: a text-mining analysis
Source: BMC Health Serv Res. 2022 Nov 28;22:1426. doi: 10.1186/s12913-022-08823-4 (PMC9703739; doi:10.1186/s12913-022-08823-4)
Supplement: Supplementary file 2 — Additional file 2. [file 12913_2022_8823_MOESM2_ESM.docx]

# Multimedia Appendix B

| Determinants of the TAM | Nr. | Questions |
| --- | --- | --- |
| Computer Self-Efficacy | 1 | What do you think of when you hear ‘digital technologies’? |
| Experience | 2 | Describe a typical working day. |
| Experience | 3 | For which activities do you use digital technologies? |
| Experience | 4 | How do you experience the impact of digital technologies on your everyday work? |
| Job Relevance | 5 | What role do digital technologies play in your work? |
| Output Quality / Result Demonstrability / | 6 | From your point of view, what are the 3 most stressful digital technologies you use? |
|  | 6.1 | Why do you experience the [technology] as stressful? |
|  | 6.2 | How does the [technology] influence your performance? |
| Output Quality / Result Demonstrability | 7 | From your point of view, what are the 3 most supporting technologies you use? |
|  | 7.1 | Why do you experience the [technology] as supporting? |
|  | 7.2 | How does the [technology] influence your performance? |
| Experience | 8 | What digital technology has been implemented recently and how did you experience this implementation? |
| Experience | 9 | Tell about a sense of achievement in working with digital technologies. |
| Computer Self-Efficacy | 10 | How do you assess your competence in dealing with digital technologies in your workplace? |
| Perceived ease of use | 11 | Can you tell me how you rate the reliability of the digital technologies provided in your workplace? |
| Computer Anxiety | 12 | How do you experience the overload caused by digital technologies in your work? |
| Computer Anxiety | 13 | To what extent are you concerned about exposing your privacy using digital technologies? |
| Management / Organizational support | 14 | Do you sometimes have to work with the digital technologies in your free time? |
| Management / Organizational support | 15 | Describe how you can separate your private life from your work due to digital technologies. |
| Management / Organizational support | 16 | How do you experience the change in your role due to digital technologies? |
| Management / Organizational support | 17 | To what extent are you interrupted in your work by digital technologies? |
| Objective Usability | 18 | Give an example of how you deal with the demands on you with regard to digital technologies. |
| Design characteristics | 19 | How do you feel about the possibility of another person being able to monitor all your performance through a digital technology? |
| User participation | 20 | What digital technologies would you like to have to better manage your work? |
